# Editorial: Postural control, exercise physiology and the balance training—type of exercises, mechanisms and insights, volume II

**DOI:** 10.3389/fphys.2024.1428692

**Published:** 2024-05-23

**Authors:** Antonino Patti, Francesco Fischetti, Fatma Nese Sahin, Antonino Bianco

**Affiliations:** ^1^ Sport and Exercise Sciences Research Unit, Department of Psychology, Educational Science and Human Movement, University of Palermo, Palermo, Italy; ^2^ Department of Basic Medical Sciences, Neuroscience and Sense Organs, University of Study of Bari, Bari, Italy; ^3^ Department of Coaching Education, Faculty of Sport Science, Ankara University, Ankara, Türkiye

**Keywords:** motor skills, evaluating balance, performance, posture, dynamic balance

Balance is a fundamental motor skill critical for human life. It is crucial for maintaining proper posture, overall well-being, and performing complex movements, especially in sports (Jaworski et al., 2023; Patti et al., 2023). Assessing the motor abilities of children is crucial for determining their developmental advancement in relation to their age. Early identification of any motor deficits facilitates timely intervention with customised programs, ensuring children acquire vital motor skills for future growth (Gallahue and Ozmun, 1998). However, it is important to acknowledge that performance, especially in children, can be influenced not only by physical and developmental factors but also by environmental variables such as stress or anxiety (Bonavolontà et al., 2021). Karadeniz et al. conducted a study including 842 primary school students, and presented their findings in this context. The present study aimed to examine the fundamental motor abilities of boys and girls aged 5–14 (Karadeniz et al., 2023). The authors noted a significant correlation between age and balancing ability, as evidenced by the increase in total KTK scores with age. The study results indicated that girls scored higher than boys. Furthermore, analyses of BMI data revealed lower balance performance among obese children, emphasising the importance of balance assessment in obese children (Karadeniz et al., 2023). The specific sports performed may affect the postural system and the development of the individual (Amato et al., 2023). Pekel et al. and Arslen et al. have reported interesting results (Pekel et al., 2023; Arslan et al.). Pekel et al. investigated the effects of a 6-week karate and basic movement training program on the balance children with congenital vision impairment, aged 10–14. Pekel et al. investigated the effects of a 6-week karate and basic movement training program on the balance of children with congenital vision impairment, aged 10–14. Their findings demonstrated significant improvements in balance among participants in the training compared to those who did not engage in any exercise. Significant differences were observed between pre- and post-test values in groups undergoing karate and basic movement training, whereas no progress was evident in the control group. These results are consistent with the study by Arslan et al., who reported the positive effects of a 10-week karate training program on the motor development of young participants (Arslan et al.). Rizzato et al. examined the crucial role of muscular strength and balance control in rugby players, specifically examining whether clenching one’s jaw with mouthguards may enhance dynamic balance and quadriceps isometric strength (Rizzato et al., 2023). Although their study suggested that clenching the jaw with a mouthguard may increase maximum strength and the rate of force development generated in the lower limbs, it did not demonstrate any improvement in the ability to maintain balance (Rizzato et al., 2023). Notably, the study by Wang and Dong Delong challenged the general view of the standing long jump as solely a measure of human muscular power, correlating with the number of fast-twitch muscle fibres and the size of the muscle’s cross-sectional area (Patti et al., 2022a; Patti et al., 2022b). Their study aims to provide a more accurate evaluation of the muscles that are more crucial from a quantitative standpoint and to better differentiate normative motions across stages (Wang and Dong). The researchers demonstrated a significant correlation between the deltoid muscle’s strength and jump performance. This highlights the need to have strong deltoid muscles in the upper limbs to improve the standing long jump distance. Furthermore, the time-series analysis revealed dynamic fluctuations in distinct muscle groups across different phases of the leap (Wang and Dong). Guo et al. explored the potential of plantar pressure analysis for assessing balance function in healthy young men. They demonstrated that visual cues, variation in step height, and foot dominance influence static imbalance during both bipedal and unipedal stances (Guo et al.). In conclusion, this second edition of our Research Topic has presented a multidisciplinary evaluation of the postural system, focusing on the impact of sports participation at both amateur and competitive levels on posture. The findings emphasise the necessity for further research in this domain, particularly regarding the development of advanced technologies and tailored assessment protocols specifically tailored to sports contexts.
